# Lack of Opsonic Antibody Responses to Invasive Infections With *Streptococcus dysgalactiae*

**DOI:** 10.3389/fmicb.2021.635591

**Published:** 2021-04-27

**Authors:** Anna Bläckberg, Therese de Neergaard, Inga-Maria Frick, Pontus Nordenfelt, Rolf Lood, Magnus Rasmussen

**Affiliations:** ^1^Division of Infection Medicine, Department of Clinical Sciences Lund, Lund University, Lund, Sweden; ^2^Skåne University Hospital, Department of Infectious Diseases, Lund, Sweden

**Keywords:** *Streptococcus dysgalactiae*, *emm* type, antibody responses, recurrent infection, bacteraemia

## Abstract

**Introduction:**

*Streptococcus dysgalactiae* can cause severe recurrent infections. This study aimed to investigate antibody responses following *S. dysgalactiae* bacteraemia and possible development of protective immunity.

**Materials and Methods:**

Patients with *S. dysgalactiae* bacteraemia in the county of Skåne between 2017 and 2018 were prospectively included. Acute and convalescent sera were obtained. All isolates were *emm* typed and enzyme-linked immunosorbent assay (ELISA) was utilised to analyse specific antibody responses to bacteria and antigens. Bactericidal- and phagocytosis assays were applied to further establish antibody function.

**Results:**

Sixteen patients with *S. dysgalactiae* bacteraemia were included of whom one had recurrent episodes of bacteraemia. Using ELISA with *S. dysgalactiae* isolates and mutants, development of IgG antibodies was demonstrated in few patients. Type-specific antibodies were demonstrated in one patient when recombinant M proteins as antigens, were applied. The type-specific serum mediated a small increase in phagocytosis but did not facilitate increased killing of the *S. dysgalactiae* isolate, carrying that M protein, in blood or by phagocytic cells.

**Conclusion:**

*S. dysgalactiae* bacteraemia sometimes results in increased levels of antibodies to the infecting pathogen. We did not find evidence that these antibodies are effectively opsonising. Apparent failure to produce opsonising antibodies might partially explain why *S. dysgalactiae* can cause recurrent invasive infections in the same host.

## Introduction

Recurrent *Streptococcus dysgalactiae* bacteraemia has been reported throughout the world, with rates of 4 to 10% following an episode with bacteraemia. In the majority of these studies, isolates carrying the same *emm* type were isolated in both episodes (Cohen-Poradosu et al., 2004; Liao et al., 2008; Rantala, 2014; Trell et al., 2016). The most common condition caused by *S. dysgalactiae* is erysipelas (also referred to as superficial cellulitis) which is an infection to the skin with high risk of recurrent infection (Inghammar et al., 2014). Recent studies have described colonisation with *S. dysgalactiae* in the perianal tract in many patients with erysipelas, even after antibiotic treatment (Eriksson, 1999; Eriksson et al., 2019). This may indicate a host-specific colonisation and a potential risk for recurrent bacteraemia.

Similar to *Streptococcus pyogenes*, *S. dysgalactiae* expresses M proteins which can confer resistance to phagocytosis and interfere with components of the coagulation system resulting in a higher persistence of bacteria in the infected tissue (Smeesters et al., 2010). In contrast to *S. dysgalactiae* there are very few reported studies of recurrent bacteraemia due to *S. pyogenes* (Rasmussen, 2011). In *S. pyogenes*, protective immunity has been attributed to antibodies against the hypervariable NH_2_-terminal part of the M protein to which Rebecca Lancefield described the development of M-type specific antibodies following *S. pyogenes* bacteraemia (Lancefield, 1959). This finding has been confirmed in later studies (Bencivenga et al., 2009). It has not been investigated if type-specific opsonic antibodies develop after *S. dysgalactiae* bacteraemia. In a mouse model of soft tissue infection with *S. dysgalactiae*, Bisno and Gaviria (1997) noted that the animals did not develop protection despite repeated episodes of infection. This was interpreted as a lack of development of protective immunity.

To avoid the action of antibodies, beta-haemolytic streptococci have developed multiple strategies, such as direct non-immune binding of antibodies through the M protein or protein G (Sjöbring et al., 1991; Courtney and Li, 2013) or enzymatic degradation of IgG (IdeS) or IgG glycans (EndoS; Collin and Olsén, 2001; Sjögren et al., 2011; von Pawel-Rammingen, 2012). These interactions are believed to help the bacteria survive in environments where opsonic antibodies are present in sufficient concentrations (Nordenfelt et al., 2012).

This study aimed to investigate the antibody response in patients with *S. dysgalactiae* bacteraemia since a lack of such a response would explain the propensity of *S. dysgalactiae* to cause recurrent infections.

## Materials and Methods

### Data Collection and Patient Inclusion

Patients with *S. dysgalactiae* bacteraemia were identified through the Laboratory for Clinical Microbiology in the county of Skåne, Sweden between 2017 and 2018. Patients were included after informed and written consent. Acute sera were obtained <5 days after admission and at follow-up 6 weeks after admission convalescent sera were obtained. Epidemiological, clinical parameters and microbiological results for each patient were collected through the medical records.

### Isolates, Species Determination and *emm* Typing

Blood isolates of *S. dysgalactiae* were collected from the Laboratory for Clinical Microbiology, Lund University Hospital Sweden and cultured on blood agar plates in 37°C and 5% CO_2_. The isolates were species determined utilising Microflex MALDI-TOF MS (Bruker, Bremen, Germany) with the direct transfer protocol (Bizzini et al., 2010) and the software FlexControl and MBT Compass 4.1 with reference database DB-8468 MSP 2019 (MALDI Biotyper; Bruker) where a score value ≥ 2.0 was needed for species determination. *emm* typing based on the sequence of the *emm* gene was performed as described at^1^. The *emm* gene sequences were compared with those in the CDC *emm* sequence database. An isogenic mutant lacking the IgG-binding protein protein G (G45ΔproteinG) was used as a control for reducing non-antigenic binding (Nitsche-Schmitz et al., 2007). G45ΔproteinG was cultivated in Todd-Hewitt broth (TH), with a final concentration of 1 μg/ml erythromycin.

### Recombinant M Protein

Fragments of the M protein, corresponding to the mature protein, from the two most common *emm* types in the study [stG2078: aa 42–514, (sequence ID WP_148848339.1) and stG62647 aa 42–457, (sequence ID WP_084917006.1)] were synthesised and ordered from GenScript. The genes were codon optimised for expression in *Escherichia coli.* The constructs, containing a *C*-terminal His-tags for simplified purification, were delivered in pET26 b vectors and expressed recombinantly in *E. coli* TUNER (DE3). Purification was conducted using Ni-affinity chromatography according to standard protocols at the Lund Protein Production Platform LP3.

### Antibody Determinations

Quantitative analysis of IgG antibodies was performed at the Department of Clinical Chemistry in Skåne, Sweden using turbidimetry. Antibodies toward IgG produce agglutinate together with the antigen. The absorbance was measured biochromatic at 340 and 700 nm, following routine clinical procedures.

### ELISA

IgG in acute and convalescent sera was quantified using enzyme-linked immunosorbent assay (ELISA). Plates were coated either with bacteria or recombinant M protein.

For coating with bacteria, the relevant isolate was cultured on blood agar plates at 37°C and 5% CO_2_. Three or four colonies were inoculated in 20 mL coating buffer (1.69 *g* Na_2_CO_3_ and 2.94 *g* NaHCO_3_ in 1 L H_2_O, pH 9.6) mixed vigorously, and dispensed into a 96-well microtitre plate Nunc Maxisorp (ThermoFisher Scientific). Bacteria were allowed to bind to the plate overnight at 37°C, 5% CO_2_, before removing the coating buffer and heat-killing the bacteria at 80°C for 15 min. Alternatively, recombinant M proteins of stG2078 and stG62647 were diluted in coating buffer to a concentration of 50 ng/mL and added into each individual well on the microtitre plate and the plate was incubated overnight at 4°C. The wells were blocked using 5% bovine serum albumin and 0.05% Tween20 in PBS (blocking buffer) and incubated at 37°C and 5% CO_2_ for 1 h. Since *S. dysgalactiae* expresses protein G which binds IgG in a non-immune manner, serum, diluted 1:100 in blocking buffer, was preincubated with IdeS, (1:40 enzyme: substrate) at 37°C and 5% CO_2_ for 50 min to generate F(ab′)_2_ and Fc fragments of IgG (Rungelrath et al., 2017). The mixture was then added into each well of a microtitre plate coated as described above. The plate was incubated at 37°C and 5% CO_2_ for 1 h and washed three times in PBST. Protein L with covalently linked HRP (GenScript), which specifically binds to the Fab-fragment of IgG, was then diluted 1:3000 (0.17 μg/ml) in blocking buffer, added to each well and the plate was incubated at 37°C and 5% CO_2_ for 1 h. This was followed by three wash steps in PBS with 0.05% Tween20. ABTS 2% 2,2′-azino-di-(3-ethylbenzthiazoline sulfonic acid) dissolved 1:20 in a substrate buffer (21 *g* citric acid monohydrate and 17.8 *g* Na_2_HPO_4_ 2 H_2_O in 1 L H_2_O, pH 4.5) was mixed in combination with 30% H_2_O_2_ and incubated in the dark in room temperature for 15–30 min. The absorbance was read in iMark Microplate reader (Bio-Rad) at 415 nm.

### Bactericidal Assay

An overnight culture of *S. dysgalactiae* bacteria was diluted in TH, 1:20, and grown to early mid-log phase (OD_620_ 0.2) and diluted to approximately 2 × 10^3^ cfu/mL in TH. Patient serum (25 μl) was mixed with freshly obtained human heparinised whole blood (125 μl) from a healthy donor and preincubated with 1U β-lactamase (β-lactamase Blend, recombinant expressed in *E. coli*, L7920, and Sigma-Aldrich) to degrade antibiotics possibly present in the patient sera. The mixtures with or without IdeS (1:40 enzyme: substrate) were incubated at 37°C and 5% CO_2_ for 20 min. Bacterial suspension (25 μl) was added to the mixture and incubation continued by gentle rotation at 37°C and 5% CO_2_ as originally described by Lancefield (Lancefield, 1957). Aliquots of the mixture were taken at different times and plated in soft agar on TH-plates. Colony forming units (cfu) were counted after overnight incubation at 37°C and 5% CO_2_. Paired acute and convalescent sera from patients were applied in the experiments. As a control, serum from the healthy donor of whole blood was utilised.

### Phagocytosis Assay

Phagocytosis assay was performed and analysed using the PAN-method as described (23). Briefly, bacteria were grown to exponential growth, heat-killed (80°C, 5 min) and stained with 5 μM Oregon Green 488-X succinimidyl ester (Invitrogen) and CypHer5E (General Electric) 7 μg/ml. Opsonisation occurred at 37°C for 30 min.

THP-1 cells (ATCC TIB-202, male) were maintained in Roswell Park Memorial Institute medium 1640 (Sigma-Aldrich) supplemented with 10% fetal bovine serum (Gibco) and 2 mM GlutaMAX (Life Technologies). The phagocytosis was performed with 100,000 cells in a final volume of 150 μl with multiplicity of prey (MOP) between 0 and 400, at 37°C or on ice as control for internalisation. Phagocytosis was haltered by putting the samples on ice for at least 15 min before data acquisition. Four independent experiments were performed.

Flow cytometric acquisition was performed using CytoFLEX (Beckman-Coulter, lasers: 488 nm, 638 nm, filters: 525/40, 660/10). At least 5,000 cells were acquired and then analysed using FlowJo version 10.2 (Tree Star), see Supplementary Material for detailed information on settings and gating strategy. THP-1 cells positive for Oregon Green-signal were defined as associating cells, and of those THP-1 cells, cells positive for CypHer5E-signal were defined as internalising cells.

Phagocytosis data were analysed using Prism 8.2.1 (GraphPad) using the PAN-template v 1.1 available at Nordenfelt Lab (zenodo version link). Normalisation for association was performed by interpolating the MFI corresponding to the MOP which evoked half of the maximal response (MOP_50_). The fluorescent signal of a prey unit was determined by measuring the MFI of free bacteria by flow cytometry. The number of prey per phagocyte, PxP, could be quantified by dividing each signal (associated: Oregon Green, internalised: CypHer5E) with the corresponding prey unit signal at pH 5. Streptococci is not typically present as a single bacterium so a prey unit most likely represents a chain.

### Statistics

For categorical data Fisher’s exact test was applied and comparisons of continuous variables were performed utilising Mann–Whitney *U* test. For comparison of paired observations Wilcoxon matched pairs signed rank test was used. Significance was defined as a *p* value ≤ 0.05. Microsoft Excel 2016 (Microsoft Corporation) was used for data collection and GraphPad Prism, version 8.2.1 (GraphPad Software) was utilised for statistical assays. In phagocytosis assay the non-parametric paired Friedman test with Dunn’s multiple comparisons test was used to test if there was a significant (alpha 0.05) difference between the episodes in association and internalisation at MOP_50_.

## Results

### Clinical Features of Patients With *S. dysgalactiae* Bacteraemia

Nineteen patients were initially included of whom two patients were excluded due to inability to obtain convalescent sera. One patient deceased prior to convalescent serum was obtained. During the study period, 2017-2018, 129 cases of *S. dysgalactiae* bacteraemia occurred in the three hospitals (Helsingborg, Lund, and Malmö) where patients were enrolled. Median age of these patients was 76 years (IQR 69-87) and 55% were male.

Sixteen patients with *S. dysgalactiae* bacteraemia were finally included in the study. The most common *emm* types were stG62647 (*n* = 5; carrying group C antigen), stG2078 (*n* = 3), and stG480 (*n* = 2; carrying group G antigen; Table 1). Table 1 summarizes the clinical characteristics of each patient. The most common focus of infection was soft tissue (69%). One patient, denoted PW had three episodes of bacteraemia with the same *emm* type during 1 year (*emm* 2078).

**TABLE 1:** Clinical features of patients with *Streptococcus dysgalactiae* bacteraemia.

***Sex/Age***	***emm type***	***Underlying disease***	***Onset to hospitalisation (days)***	***Length of stay (days)***	***Focus of infection***
F55	Non-typeable	Previous malignancy (breast). Lymphoedema	1	9	Erysipelas (arm)
F82	stG62647	CF	1	14	Post op wound
F77	stG480	COPD. HF. Previous malignancy (endometrial)	1	8	Erysipelas (leg)
M79	stG62647	HF. Previous malignancy (prostate)	1	5	Erysipelas (leg)
M79	stG2078	HF. CKD	<1	14	Unknown
M80	stG507	Alcoholism. Cirrhosis	3	7	Erysipelas (leg). Pneumonia
M75	stG62647	HF. Previous malignancy (prostate)	1	16	Erysipelas (abdominal). Abscess (back)
M77	stG62647	Liver disease. HF. Malignancy (rectal and urinary bladder)	1	7	Unknown
M66	stG6	DM. CVI	<1	9	Erysipelas (leg)
F84	stC74a	DM. CF. HF	1	11	Pyelonephritis
M81	stG480	CVI. HF	<1	11	Erysipelas (leg)
F59	stG10	Obesity	<1	5	Erysipelas (leg)
M65	stG62647	COPD. Malignancy (rectal)	<1	20	Unknown
F80^∗^	stG2078	HF.CKD. Previous malignancy (ovarian). Lymphoedema	<1	7	Erysipelas (leg)
F80^∗^	stG2078	HF. CKD. Previous malignancy (ovarian). Lymphoedema	<1	7	Erysipelas (leg)
F86	stG485	DM. HF. Previous malignancy (breast). Lymphoedema	<1	13	Erysipelas (leg)
M82	stG643	DM. CVI	<1	7	Erysipelas (leg). Pneumonia

CF, cognitive failure; COPD, chronic obstructive pulmonary disease; HF, heart failure; CKD, chronic kidney disease; DM, diabetes mellitus; and CVI, cerebrovascular insult.*Same patient.

### Lack of Seroconversion in Convalescent Sera Against G45ΔproteinG

**FIGURE 1:**
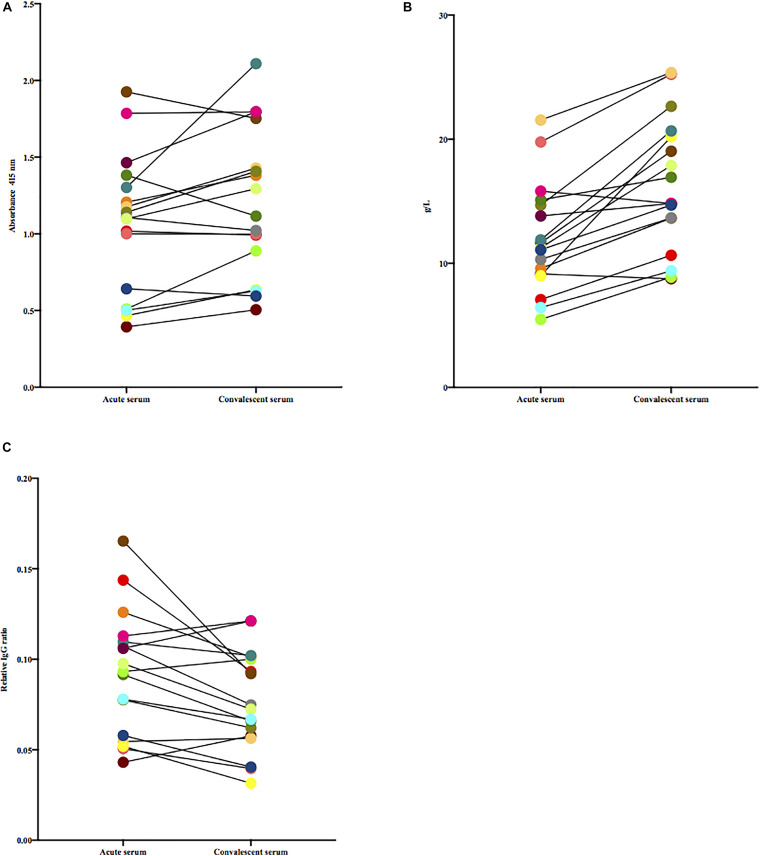
**(A)** Paired acute and convalescent sera from patients were tested for the presence of antibodies toward G45ΔproteinG. Wilcoxon matched-pairs signed-rank test was used for comparisons. **(B)** Total levels of IgG in paired acute and convalescent sera of included patients, quantified by routine clinical procedures, were tested for difference using Wilcoxon matched-pairs signed-rank test. **(C)** Paired acute and convalescent sera from patients were tested for the presence of antibodies toward G45ΔproteinG, adjusted for total IgG. Wilcoxon matched-pairs signed-rank test was used to test if difference was statistically significant.

Paired acute and convalescent sera from patients were tested for the presence of antibodies against *S. dysgalactiae*. To avoid the non-immune binding of IgG to protein G, a mutant *S. dysgalactiae* strain lacking protein G (G45ΔproteinG) was used to coat the plates. There were no significant differences in levels of absorbance toward G45ΔproteinG between acute and convalescent patient sera, [Wilcoxon matched-pairs signed-rank test, (*p* = 0.052)] (Figure 1A). Total levels of IgG in serum, however, were significantly higher in convalescent than in acute sera, [Wilcoxon matched-pairs signed-rank test, (*p* < 0.0001)] (Figure 1B). Figure 1C, displays absorbance levels from the ELISA (Figure 1A) divided by the concentration of total IgG (Figure 1B). Adjusting for total IgG-levels, acute sera displayed a stronger signal in ELISA compared to convalescent sera [Wilcoxon matched-pairs signed-rank test, (*p* = 0.008)].

### Increased Antibody Levels Against the Patient-Specific *S. dysgalactiae* Isolate

**FIGURE 2:**
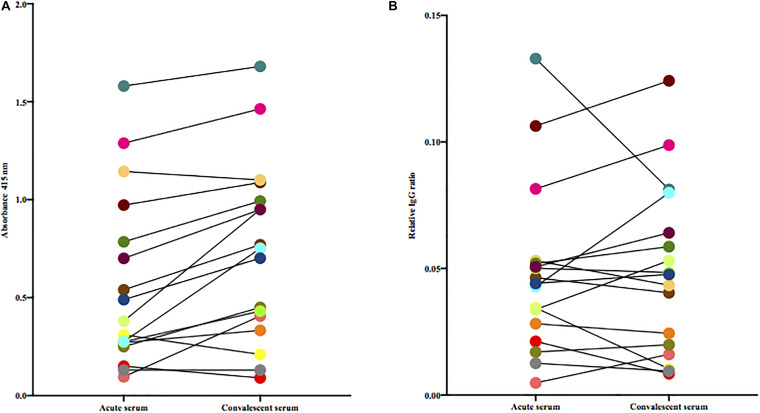
**(A)** Paired acute and convalescent sera from patients were tested for the presence of antibodies toward the bacterium isolated from the same patient. Statistical difference was assessed using Wilcoxon matched-pairs signed-rank test. **(B)** Paired acute and convalescent sera from patients were tested for the presence of antibodies toward the patient’s own bacterial isolate adjusted for total IgG (Wilcoxon matched-pairs signed-rank test).

Since the M protein is believed to be a major antigenic structure of *S. dysgalactiae* and is variable between isolates, we next tested the levels of antibodies in acute and convalescent sera against the patient-specific isolate. Using this experimental set-up, we observed a median increase of 0.18 in absorbance between acute and convalescent sera [Wilcoxon matched-pairs signed rank test, (*p* = 0.0006)] (Figure 2A). However, this difference was not observed when adjusting for total levels of IgG (Figure 2B).

### Type Specific and Cross-Reactivity Among Different *emm* Types

To investigate the role of the M protein in the immune response to *S. dysgalactiae*, we analysed the binding of serum IgG to recombinant M proteins, representing the two major *emm* types in the study (e.g., stG2078 and stG62647), both in acute and convalescent sera from some of the patients (Table 2). To gain insight relating to cross-reactivity between M proteins, serum was pre-incubated with either stG2078, stG62647, or PBS (control) before the interaction between antibodies in serum and the immobilised M protein was measured. Antibody binding was often increased in the convalescent serum, as compared to the acute serum. Patients infected with *emm* types other than stG2078 and stG62647 showed no proof of major IgG reactivity with any of the two recombinant M proteins.

**TABLE 2:** Absorbance 415 nm, patient sera and coating with recombinant M proteins.

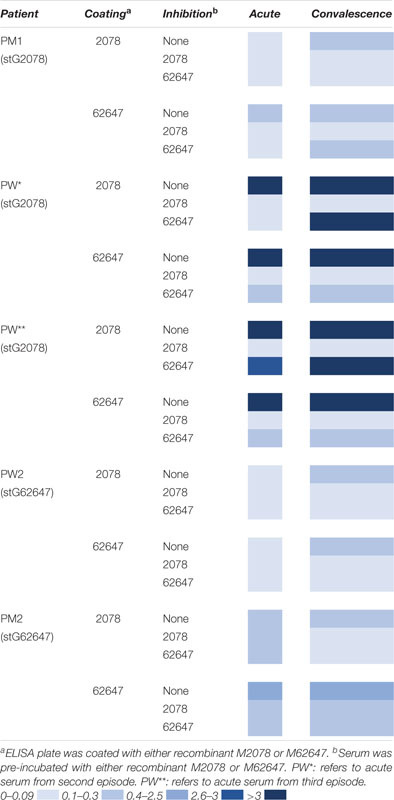

### Seroconversion in PW

The patient, PW, had three episodes of bacteraemia with stG2078 during the study period and we had access to serum drawn before the second episode and after the third episode. A high level of antibody binding toward M2078 and M62647 was observed in both sera. In the serum drawn before the second episode this reactivity could be inhibited either by the addition of soluble M2078 or M62647 to the reaction (Table 2) indicating that this serum contained antibodies directed toward epitopes shared between the two M proteins. In the serum drawn after the third episode, however, the reactivity toward M2078 was inhibited by M2078 but not by M62647. This indicates that type-specific antibodies had been formed in this patient.

### Lack of Bactericidal Activity of Type-Specific Antibodies

**FIGURE 3:**
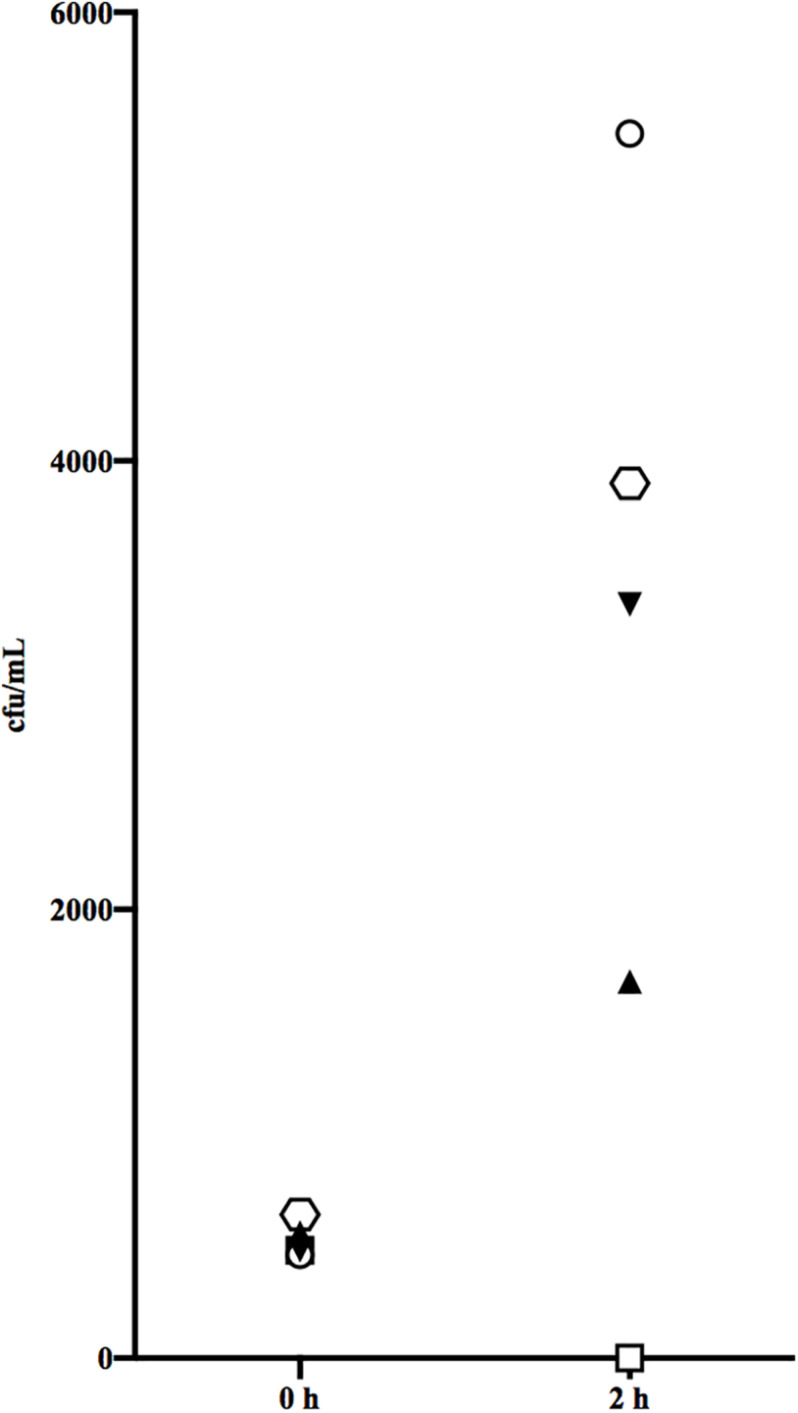
Bactericidal assay measured the functional activity of antibodies in the acute serum from the third episode and convalescent serum. Acute serum from episode 3 (triangle), with IdeS added (inverted triangle) and convalescent serum (hexagon), with IdeS added (circle) and no beta lactamase added (quadrangle). The result presented is from one representative experiment.

The paired sera from PW and the formation of type-specific antibodies were next tested for their ability to confer killing of the stG2078 from the same patient in non-immune blood from a healthy donor. Since the sera contained antibiotics, they were first treated with β-lactamase. Figure 3 summarises results from the bactericidal assay. Unexpectedly, more bacteria were retrieved after incubation of the blood with serum containing type-specific antibodies (convalescent serum) than after incubation with serum lacking such antibodies. Preincubation with IdeS, cleaving and inactivating IgG, resulted in increased levels of cfu/mL in both acute and convalescent serum.

### Phagocytosis

**FIGURE 4:**
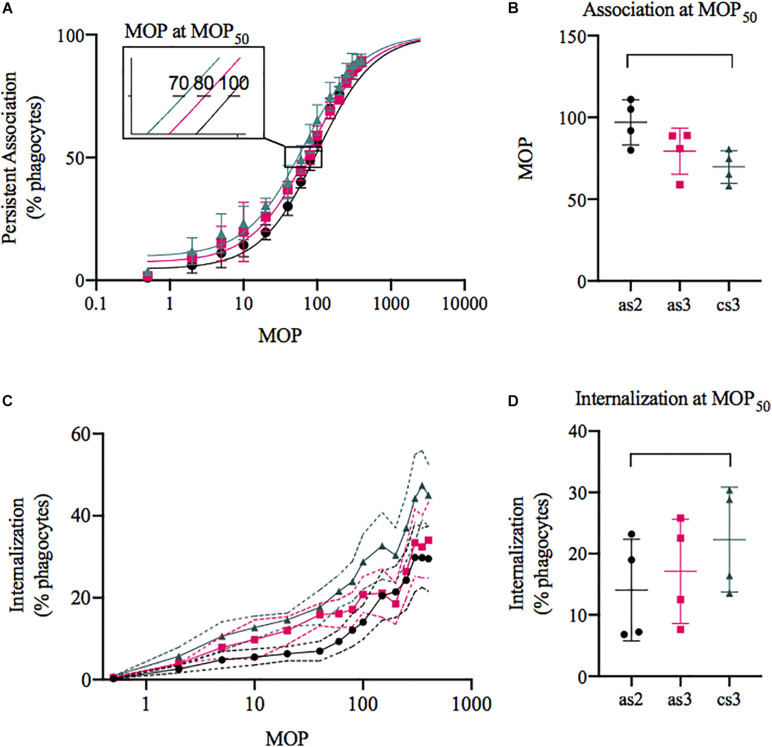
Phagocytosis of *S. dysgalactiae* isolated from the patient with three bacteraemia episodes with seroconversion. The bacteria were opsonised with the acute serum from episode 2 (black, as2), 3 (pink, as3), or the convalescent serum (green, cs3) and then incubated with the THP-1 cells. Data was acquired through flow cytometry and is presented as mean ± SD (**C:** visualised with SEM), *n* = 4. **(A,C)** The percentage of THP-1 cells associated **(A)** or internalising **(C)** with at least one prey plotted on the *y*-axis and the multiplicity of prey (MOP) on the *x*-axis. **(B)** The MOP to evoke 50% association (MOP_50_) was 70 ± 10.0, 79 ± 14.1, and 97 ± 13.8 (cs3, as3, and as2). **(D)** At MOP_50_ the percentage of THP-1 cells internalising was 14 ± 8.3, 17 ± 8.5, and 22 ± 8.6 (as2, as3, and cs3).

Serum from the patient, PW, which showed seroconversion between the second and the third episode, was assessed with respect to phagocytosis with an in-depth flow cytometric assay that takes into account both phagocyte association and internalisation (de Neergaard et al., 2019). Figure 4A visualizes the association capacity of the phagocytes with the percentage of phagocytes interacting (adhered or internalised) with at least one bacterium on the *y*-axis and the MOP on the *x*-axis. An increase in association capacity results in a left shift of the curves. This can be quantified by comparing the MOP needed to reach 50% association (MOP_50_), which resulted in a small decrease from 97 ± 14 (SD) in average to 70 ± 10 (SD) with a significant increase in phagocyte association (*p* = 0.04) between the first and third serum (Figure 4B). The same trend can be seen in the percentage of phagocytes internalising at least one prey (Figure 4C) and at MOP_50_ there is a small significant increase (*p* = 0.04) from 14 ± 8.3% to 22 ± 8.6% (SD) internalisation between the first and third serum (Figure 4D). We also wanted to see if there was a difference once a phagocyte had internalised a bacterium. The number of prey associated per phagocyte decreases from on average 14 ± 3.1 to 11 ± 2.2 (SD; Supplementary Figure 1A) between the first and third serum and the number of prey internalised per phagocyte from 7.5 ± 1.6 to 7.0 ± 0.8 (SD; Supplementary Figure 1B). There was no significant difference (*p* = 0.07 association, *p* = 0.1 internalisation) between the sera, but the data indicate a decrease in association and internalisation ability of internalising phagocytes. Taken together with the population assessment above, this means that with the serum containing type-specific antibodies, more phagocytes associate with bacteria, leading to an overall small increase in phagocytosis, but on an individual level they do not become better at associating with or internalising their prey.

## Discussion

Between 4 and 10% of persons with *S. dysgalactiae* bacteraemia experience a reoccurrence, most often within 6 months after the first episode. Previously, several studies have described development of type-specific antibodies after *S. pyogenes* bacteraemia which are believed to protect from future infections with the same *S. pyogenes* type. However, the humoral immune response to *S. dysgalactiae* bacteraemia has been poorly described. The results from this study indicate that the development of type-specific antibodies following *S. dysgalactiae* bacteraemia does not occur in a majority of patients. Only in one patient, we observed the formation of type-specific antibodies that only slightly increased the overall phagocytosis by increasing the portion of phagocytes involved but failed to improve the individual phagocyte’s ability to phagocytise and mediate killing in our experimental systems. A lack of development of protective antibodies during invasive infection with *S. dysgalactiae* could, at least in part, explain the tendency of *S. dysgalactiae* bacteraemia to recur. In addition to lack of protective immunity, persistent colonisation, which has been observed for *S. dysgalactiae* recently (Trell et al., 2019), may also be an important factor for increased risk of recurrence.

Streptococcus *dysgalactiae* expresses protein G which binds IgG in a non-immune manner through interactions both with the heavy and light chain of IgG (Erntell et al., 1988). Protein G makes the study of immune responses toward *S. dysgalactiae* difficult since this protein will absorb IgG which will be detected in the ELISA assays. We employed several strategies to circumvent this problem. The first strategy employed was to use a mutated *S. dysgalactiae* strain lacking protein G. In such a system, only antibodies reacting with conserved *S. dysgalactiae* proteins will be detected. Our second strategy was therefore to use the bacterium isolated from every given patient together with serum from the same patient and cleaving the IgG into F(ab′)_2_ and Fc fragments using IdeS. With this strategy, we detected the bacterial associated Fab-fragments by the light chain-binding protein L as Fab-specific antibodies would again be absorbed by protein G expressed by the bacteria. Moreover, in both systems, we controlled for the total IgG concentrations in the serum. The third strategy employed was to test for antibodies reacting with recombinant M proteins. Importantly, we used two M proteins which have both shared, and type specific epitopes. Thus, by competition-experiments we could determine if antibodies reacted with conserved or type-specific epitopes. Altogether, data from different experimental systems, indicated a development of antibodies following invasive *S. dysgalactiae* infection.

Despite that the patient PW apparently developed type-specific antibodies we could not detect any increased bacterial killing of bacteria in human blood with the addition of patient serum. The assay employed has previously been used for *S. pyogenes* and is technically difficult and highly dependent on the blood donor and has high interexperimental variation. Therefore, we wanted to further study if the type-specific antibodies increased the ability of phagocytes to bind and internalise *S. dysgalactiae* bacteria using high-sensitivity phagocytosis assessment (de Neergaard et al., 2019). In the phagocytosis assay we observed a small increase in the proportion of cells that associated with and internalised serum-incubated bacteria, but with no individual enhancement of phagocytic ability. This points to the existence of some agglutinating or opsonising antibodies in the seroconverted patient. However, the combination of lack of efficient killing and overall small effects on phagocytosis indicates that the type-specific antibodies either are being neutralised by the bacteria or are present in too low concentration to promote phagocytic-based immunity.

Prophylaxis with penicillin is an option in recurrent erysipelas and, considering the risk after recurrent *S. dysgalactiae* bacteraemia, prophylaxis may also be used in this condition. We believe that identification of clinical risk factors for recurrence should be used to determine which patients who would benefit from prophylaxis. Our results indicate that patients with recurrent infections might develop type-specific antibodies but the role of these antibodies in protection against future recurrent infections needs further study.

Our study has obvious limitations. Our sample size, encompassing 16 patients with paired acute and convalescent sera, is small. Generalisations based on our results should therefore be made only with great care. In addition, other aspects of the immune response antibody production toward *S. dysgalactiae*, were not investigated in our study and could potentially be very important.

## Conclusion

Development of type specific antibodies occurs only in a minority of patients following *S. dysgalactiae* bacteraemia, but we found no evidence that such antibodies are protective. Lack of opsonising antibodies may help to explain occurrence of recurrent invasive infections with *S. dysgalactiae* in the same host.

## Data Availability Statement

The raw data supporting the conclusions of this article will be made available by the authors, without undue reservation.

## Ethics Statement

The studies involving human participants were reviewed and approved by the regional ethics committee of Lund University, (2016/939). The patients/participants provided their written and oral informed consent to participate in this study.

## Author Contributions

AB and MR prepared the study design, performed statistical analyses, and drafted the manuscript. AB performed bactericidal assays and experiments. I-MF advised continuously in experiments and projects. TN performed experiments with phagocytosis assay. RL aided in study design and advising in experiments. PN assisted in experiment and design of phagocytosis assay. All authors contributed to the article and approved the submitted version.

## Conflict of Interest

The authors declare that the research was conducted in the absence of any commercial or financial relationships that could be construed as a potential conflict of interest.

## Footnotes

1 http://www.cdc.gov/streplab

## Supplementary Material

Supplementary Figure 1 Phagocytosis of *S*. *dysgalactiae* isolated from the patient with three bacteraemia episodes with seroconversion. The bacteria were opsonised with the acute serum from episode 2 (black, as2), 3 (pink, as3), or the convalescent serum (green, cs3) and then incubated with the THP-1 cells. Data was acquired through flow cytometry and is presented as mean ± SD, *n* = 4. The average number of prey associated **(A)** 14.4 ± 3.1, 10.8 ± 3.7, and 11.0 ± 2.2 SD (both adhered and internalised) and internalised **(B)** 7.5 ± 1.6, 6.4 ± 1.5, and 7.0 ± 0.8 per internalising phagocyte at MOP_50_ (as2, as3, and cs3).
