# Supplementary material for: Lack of Opsonic Antibody Responses to Invasive Infections With Streptococcus dysgalactiae
Source: Front Microbiol. 2021 Apr 27;12:635591. doi: 10.3389/fmicb.2021.635591 (PMC8111088; doi:10.3389/fmicb.2021.635591)
Supplement: Supplementary file 1 [file Data_Sheet_1.DOCX]

**Supplementary material**

**Phagocytosis assay**

*Settings and gating strategy for phagocytosis assay*

Threshold was set at FSC-H 70 000 for phagocytosis and for bacteria FSC-H 2000 and SSC-H 2000. Gain was kept at 3 for FITC and 265 for APC. The THP-1 cells were gated on forward (FSC) and side scatter (SSC) height, then doublets were excluded by gating on FSC-H versus FSC-A. Free bacteria were gated on side scatter and as positive for Oregon Green signal, then then doublets were excluded by gating on FSC-H versus FSC-A.
