# Supplementary figures and images for: Lack of Opsonic Antibody Responses to Invasive Infections With Streptococcus dysgalactiae
Source: Front Microbiol. 2021 Apr 27;12:635591. doi: 10.3389/fmicb.2021.635591 (PMC8111088; doi:10.3389/fmicb.2021.635591)

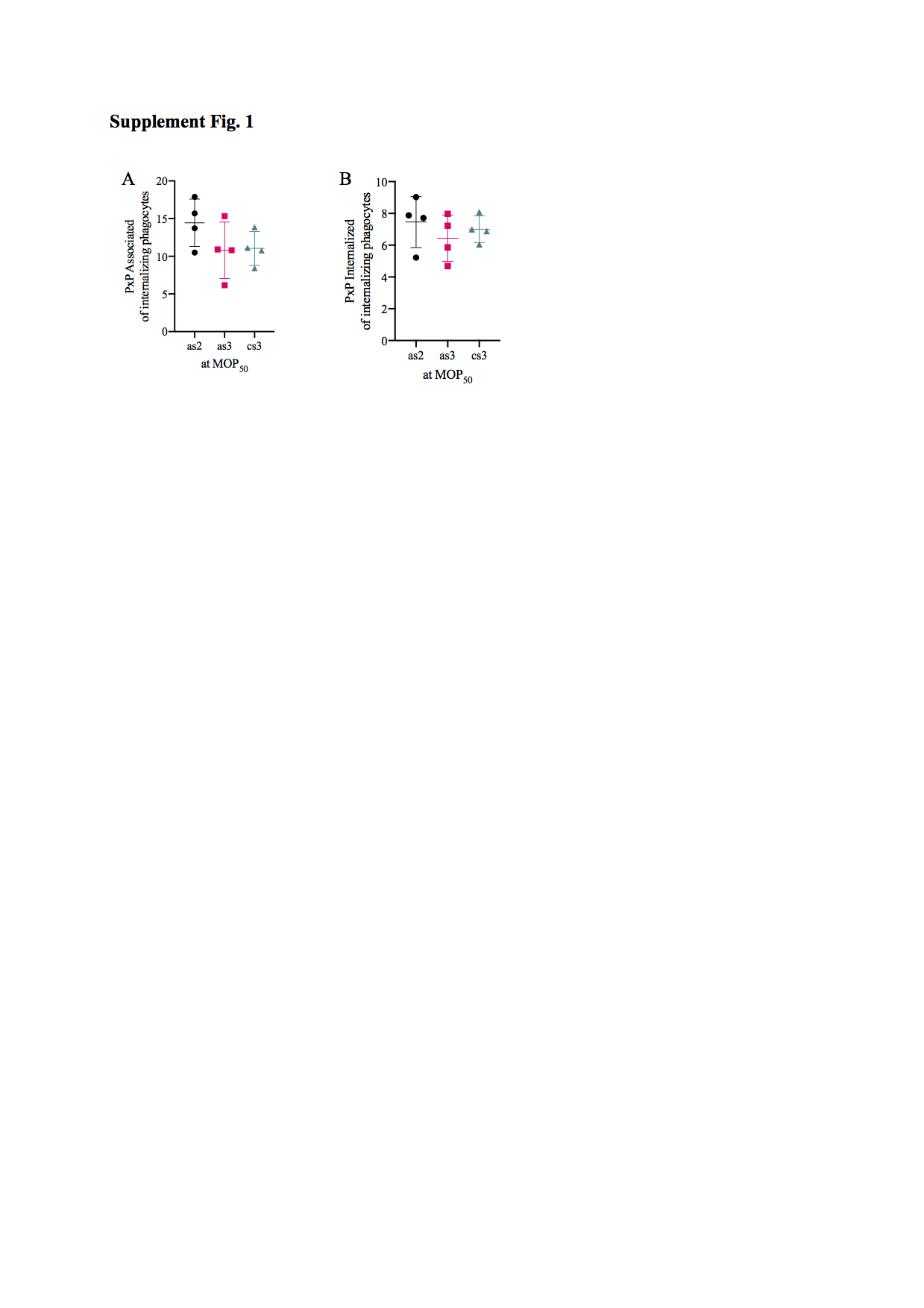

Supplement: Supplementary Figure 1 — Phagocytosis of S. dysgalactiae isolated from the patient with three bacteraemia episodes with seroconversion. The bacteria were opsonised with the acute serum from episode 2 (black, as2), 3 (pink, as3), or the convalescent serum (green, cs3) and then incubated with the THP-1 cells. Data was acquired through flow cytometry and is presented as mean ± SD, n = 4. The average number of prey associated (A) 14.4 ± 3.1, 10.8 ± 3.7, and 11.0 ± 2.2 SD (both adhered and internalised) and internalised (B) 7.5 ± 1.6, 6.4 ± 1.5, and 7.0 ± 0.8 per internalising phagocyte at MOP50 (as2, as3, and cs3). [file Image_1.tiff]
